# Analysis of Serum Advanced Glycation Endproducts Reveals Methylglyoxal-Derived Advanced Glycation MG-H1 Free Adduct Is a Risk Marker in Non-Diabetic and Diabetic Chronic Kidney Disease

**DOI:** 10.3390/ijms24010152

**Published:** 2022-12-21

**Authors:** Naila Rabbani, Antonysunil Adaikalakoteswari, James R. Larkin, Sianna Panagiotopoulos, Richard J. MacIsaac, Dennis K. Yue, Gregory R. Fulcher, Matthew A. Roberts, Merlin Thomas, Elif Ekinci, Paul J. Thornalley

**Affiliations:** 1 Department of Basic Medical Science, College of Medicine, QU Health, Qatar University, Doha P.O. Box 2713, Qatar; 2 Clinical Sciences Research Laboratories, Warwick Medical School, University of Warwick, University Hospital, Coventry CV2 2DX, UK; 3 Endocrine Centre, Austin Health, The University of Melbourne, West Heidelberg, VIC 3084, Australia; 4 Department of Endocrinology & Diabetes, St Vincent’s Hospital Melbourne, Fitzroy, VIC 3065, Australia; 5 Australian Centre for Accelerating Diabetes Innovations, School of Medicine, University of Melbourne, Parkville, VIC 3052, Australia; 6 Diabetes Centre, Royal Prince Alfred Hospital, Camperdown, NSW 2050, Australia; 7 Department of Diabetes, Endocrinology & Metabolism, Royal North Shore Hospital, St Leonards, NSW 2065, Australia; 8 Eastern Health Clinical School, Monash University, Box Hill, VIC 3128, Australia; 9 Department of Diabetes, Monash University, Melbourne, VIC 3004, Australia; 10 Diabetes Research Center, Qatar Biomedical Research Institute, Hamad Bin Khalifa University, Qatar Foundation, Doha P.O. Box 34110, Qatar

**Keywords:** chronic kidney disease, diabetes, glycation, methylglyoxal, estimated glomerular filtration rate

## Abstract

Accumulation of advanced glycation endproducts (AGEs) is linked to decline in renal function, particularly in patients with diabetes. Major forms of AGEs in serum are protein-bound AGEs and AGE free adducts. In this study, we assessed levels of AGEs in subjects with and without diabetes, with normal renal function and stages 2 to 4 chronic kidney disease (CKD), to identify which AGE has the greatest progressive change with decline in renal function and change in diabetes. We performed a cross-sectional study of patients with stages 2–4 CKD, with and without diabetes, and healthy controls (*n* = 135). Nine protein-bound and free adduct AGEs were quantified in serum. Most protein-bound AGEs increased moderately through stages 2–4 CKD whereas AGE free adducts increased markedly. Methylglyoxal-derived hydroimidazolone MG-H1 free adduct was the AGE most responsive to CKD status, increasing 8-fold and 30-fold in stage 4 CKD in patients without and with diabetes, respectively. MG-H1 Glomerular filtration flux was increased 5-fold in diabetes, likely reflecting increased methylglyoxal glycation status. We conclude that serum MG-H1 free adduct concentration was strongly related to stage of CKD and increased in diabetes status. Serum MG-H1 free adduct is a candidate AGE risk marker of non-diabetic and diabetic CKD.

## 1. Introduction

Increased formation and accumulation of advanced glycation endproducts (AGEs) have been linked to the decline in renal function and the development of chronic kidney disease (CKD), particularly in patients with diabetic mellitus [1]. AGEs are formed by the degradation of glucose-derived early-stage glycation adducts, fructosamines, by protein glycation by reactive dicarbonyl metabolites, glyoxal, methylglyoxal (MG) and 3-deoxyglucosone (3-DG), and other processes [2]. The effect of AGEs on renal function may be exacerbated in diabetes where formation of AGEs is enhanced by the hyperglycemia-related increase in fructosamine and dicarbonyl metabolite AGE precursors [2,3]. Major AGEs formed from the degradation of the fructosamine, N_ε_-fructosyl-lysine, and protein glycation by MG are N_ε_-carboxymethyl-lysine (CML) and arginine-derived hydroimidazolone MG-H1, respectively [4,5]. Pentosidine, a widely studied AGE formed mainly from pentose sugar precursors [6], is a minor glycation-derived protein crosslink related to activity of the pentosephosphate pathway [7,8]. Measurement of AGEs in serum may provide risk markers for improved clinical diagnosis of CKD in patients with and without diabetes. The relative clinical utility of different AGEs is not clear [9,10], specifically, which AGE has the greatest variation with change in estimated glomerular filtration rate (eGFR) from normal renal function to stage 4 CKD, and which has characteristic values in patients with and without diabetes.

**Figure 1:**
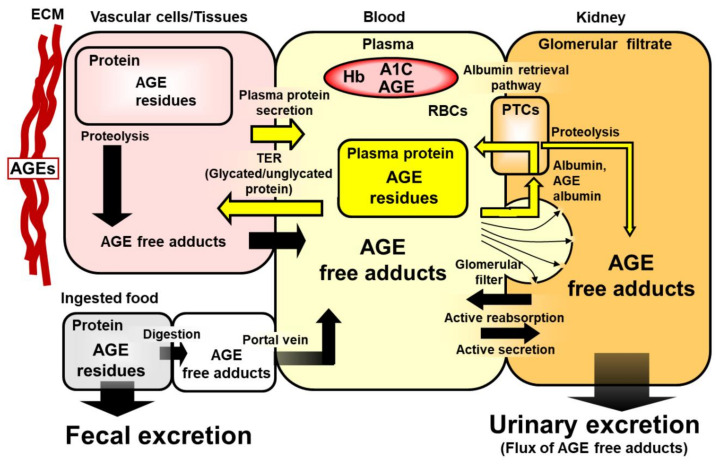
Schematic representation of formation, physiological processing and metabolic transit of protein AGEs in clinical metabolism. Block yellow arrows show movement of plasma protein out of and in to the plasma compartment by TER and tissue secretion, respectively, and glomerular filtration of AGE modified albumin and AGE free adducts with major recovery of albumin into the renal venous circulation by the albumin retrieval pathway and minor degradation by PTC proteolysis. Abbreviations: A1C, glycated hemoglobin HbA_1c_; AGE, advanced glycation endproduct; ECM, extracellular matrix; Hb, hemoglobin; PTC, proximal tubular epithelial cells; and TER, transcapillary escape rate.

In serum, AGEs are mainly present as AGE residues in proteins and glycated amino acids, referred to as “protein-bound AGEs” and AGE free adducts, respectively [1,2]. The level of protein-bound AGEs in serum reflects the steady-state balance between the rate of AGE formation in mostly the vasculature during the lifespan of the serum protein and degradation of the AGE-modified protein by cellular uptake and proteolysis. Serum protein AGE residues have low renal clearance and a biodistribution limited to that of the protein on which they are formed. The level of serum protein-bound AGEs may be influenced in CKD by changes in plasma concentration of glycating agents and leakage of albumin through the glomerular filter and albuminuria [11,12]. Serum AGE free adducts are formed endogenously by cellular proteolysis and released into plasma for urinary excretion [1,2]. There is also an exogenous contribution from intestinal absorption of AGE free adducts from digested glycated proteins in food [13,14]. The latter contribution may be minimised by collecting blood samples in the fasting state at which time most AGE free adducts absorbed from food have been cleared by glomerular filtration and accumulated in urine. AGE free adducts are small molecules of <500 Da with usually high renal clearances and are the major form by which AGEs are cleared from the body [3,15,16]. AGE free adducts accumulate profoundly in plasma with marked decline or loss of renal function, as evidenced in studies of clinical endstage renal disease and experimental bilateral nephrectomy [15,17]. Serum AGE free adducts are therefore expected to accumulate progressively with increase of CKD stage and inversely to decline in renal function [1] (Figure 1).

The aim of this study was to assess the variation in serum protein-bound and AGE free adduct levels in subjects with renal function range from normal to stage 4 CKD, with and without diabetes, with a working hypothesis that AGE free adducts accumulate to a greater extent than protein-bound AGEs as renal function declines, and AGEs with greatest increase in formation in diabetes accumulate to a higher level in patients with diabetes, compared to those without diabetes, for each stage of CKD studied.

## 2. Results

### 2.1. Subject Characteristics

There were 135 subjects (79 male, 56 female) recruited for this study: 73 without diabetes and 62 with diabetes (6 type 1 diabetes, 56 type 2 diabetes; 23 had diabetic retinopathy co-morbidity). Classified by CKD status, there were 19 subjects with normal renal function and 35, 45 and 36 with stages 2, 3 and 4 CKD, respectively. Most of the subjects were elderly, except those with diabetes with normal renal function. Of the remaining subjects, 68% were over 65 years of age. In subjects with and without diabetes, eGFR correlated negatively with age (r = −0.62, *p* < 0.001 and −0.36, *p* < 0.01, respectively)—Table 1.

### 2.2. Serum Protein-Bound AGEs

**Figure 2:**
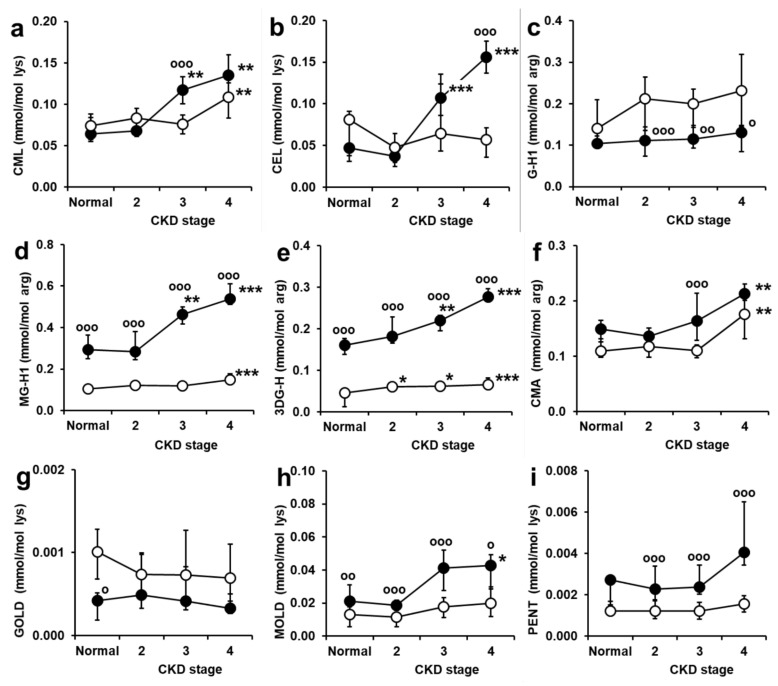
Serum protein-bound AGEs in subjects with normal renal function and stages 2–4 chronic kidney disease. (**a**) N_ε_-Carboxymethyl-lysine CML, (**b**) N_ε_-(1-Carboxyethyl)lysine CEL, (**c**) Glyoxal-derived hydroimidazolone G-H1, (**d**) Methylglyoxal-derived hydroimidazolone MG-H1, (**e**) 3-Deoxyglucosone-derived hydroimidazolone 3DG-H, (**f**) N_ꞷ_-carboxymethylarginine CMA, (**g**) Glyoxal-derived lysine dimer GOLD, (**h**) Methylglyoxal-derived lysine dimer MOLD, (**i**) Pentosidine PENT. Key: ○-○, subjects without diabetes; ●-●, subjects with diabetes. Data are median (lower–upper quartile), n = 7–29. Significance: *. ** and ***, *p* < 0.05, *p* < 0.01 and *p* < 0.001 with respect to subjects with normal renal function of the same diabetes status. o, oo and ooo, *p* < 0.05, *p* < 0.01 and *p* < 0.001 with respect to subjects without diabetes of the same stage of CKD. Significance for 4 group comparison of normal renal function and stages 2–4 CKD, without and with diabetes (*Kruskal-Wallis*) were highly significant (*p* < 0.001) except subjects without diabetes—CEL, G-H1, GOLD and pentosidine (*p* > 0.05), MOLD (*p* < 0.05) and 3DG-H (*p* < 0.01), and subjects with diabetes—GOLD and MOLD (*p* > 0.05).

Levels of protein-bound AGEs are presented in Figure 2. For N_ε_-carboxymethyl-lysine CML, N_ε_-(1-carboxyethyl)lysine CEL, glyoxal-derived hydroimidazolone G-H1, N_ꞷ_-carboxymethylarginine CMA, glyoxal-derived lysine dimer GOLD, methylglyoxal-derived lysine dimer MOLD, and pentosidine, levels were relatively low (<0.2 mmol/mol arg or lys) with limited, up to 2-fold, increase at stage 4 CKD and often no clearly discernible difference in levels for subjects with and without diabetes. For protein-bound methylglyoxal-derived hydroimidazolone MG-H1 and 3-deoxyglucosone-derived hydroimidazolone 3DG-H, there was 3–5 fold higher levels in subjects with diabetes, compared to subjects without diabetes, and increases from normal renal function to stage 4 CKD of +41% and *ca.* 3-fold for MG-H1 and +44% and *ca.* 5-fold for 3DG-H in subjects without and with diabetes, respectively.

In correlation analysis, there were no significant correlations of protein-bound AGEs with age of subjects without diabetes, whereas protein-bound CEL and pentosidine correlated positively with age of subjects with diabetes. There was no correlation of protein-bound AGEs with A1C in subjects with diabetes. Protein-bound CML, MG-H1 and 3DG-H correlated negatively with eGFR in both subjects with and without diabetes; CMA and MOLD correlated negatively with eGFR in non-diabetic subjects and CEL and pentosidine correlated negatively with eGFR in subjects with diabetes (r = −0.31 to −0.52; Supplementary Data, Table S1).

### 2.3. Serum AGE Free Adducts

**Figure 3:**
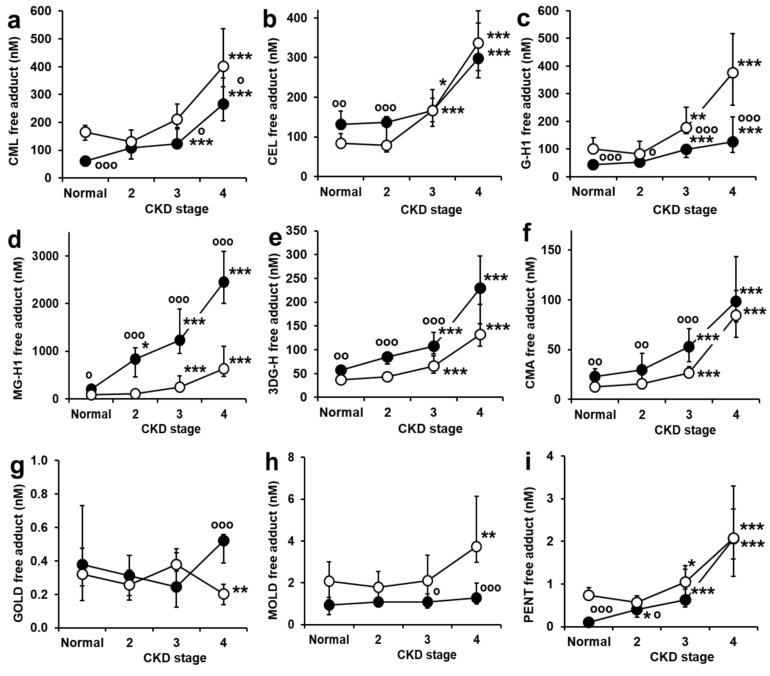
Serum AGE free adducts in subjects with normal renal function and stages 2–4 chronic kidney disease. (**a**) N_ε_-Carboxymethyl-lysine CML, (**b**) N_ε_-(1-Carboxyethyl)lysine CEL, (**c**) Glyoxal-derived hydroimidazolone G-H1, (**d**) Methylglyoxal-derived hydroimidazolone MG-H1, (**e**) 3-Deoxyglucosone-derived hydroimidazolone 3DG-H, (**f**) N_ꞷ_-carboxymethylarginine CMA, (**g**) Glyoxal-derived lysine dimer GOLD, (**h**) Methylglyoxal-derived lysine dimer MOLD, (**i**) Pentosidine. Key: ○-○, subjects without diabetes; ●-●, subjects with diabetes. Data are median (lower–upper quartile), n = 7–29. Significance: *. ** and ***, *p* < 0.05, *p* < 0.01 and *p* < 0.001 with respect to subjects with normal renal function of the same diabetes status; and o and ooo, *p* < 0.05 and *p* < 0.001 with respect to subjects without diabetes of the same stage of CKD. Significance for 4 group comparison of normal renal function and stages 2–4 CKD, without and with diabetes (*Kruskal-Wallis*) were highly significant (*p* < 0.001) except for GOLD and MOLD in subjects with diabetes (*p* > 0.05).

Serum concentrations of AGE free adducts are presented in Figure 3. Most serum free adducts increased markedly through stages 2–4 CKD, except for GOLD and MOLD. Increases were from 3- to 30-fold, depending on the AGE and diabetes status. The AGE free adduct of highest concentration, greatest progressive increase in concentration to stage 4 CKD and higher in subjects with diabetes at all stages of CKD, was MG-H1. Median serum MG-H1 free adduct concentration of subjects with normal renal function without diabetes was 82 nM, increased 2.5-fold in patients with diabetes and normal renal function, and increased progressively with stage of CKD to 8- and 30-fold increases in patients with stage 4 CKD, without and with diabetes, respectively. Surprisingly, CML and pentosidine free adducts were not consistently increased in patients with diabetes, and pentosidine free adduct was 7-fold higher in non-diabetic subjects with normal renal function than in subjects with diabetes.

In correlation analysis, serum AGE free adducts did not correlate with age in subjects without diabetes whereas all AGE free adducts except for GOLD and MOLD correlated with age in subjects with diabetes (r = 0.38–0.58). All AGE free adducts except for GOLD and MOLD correlated negatively with eGFR in both subjects with and without diabetes (r = −0.58 to −0.81). There was no correlation of serum AGE free adducts with A1C in subjects with diabetes (Supplementary Data, Table S1).

### 2.4. AGE Free Adduct Glomerular Filtration Flux

**Figure 4:**
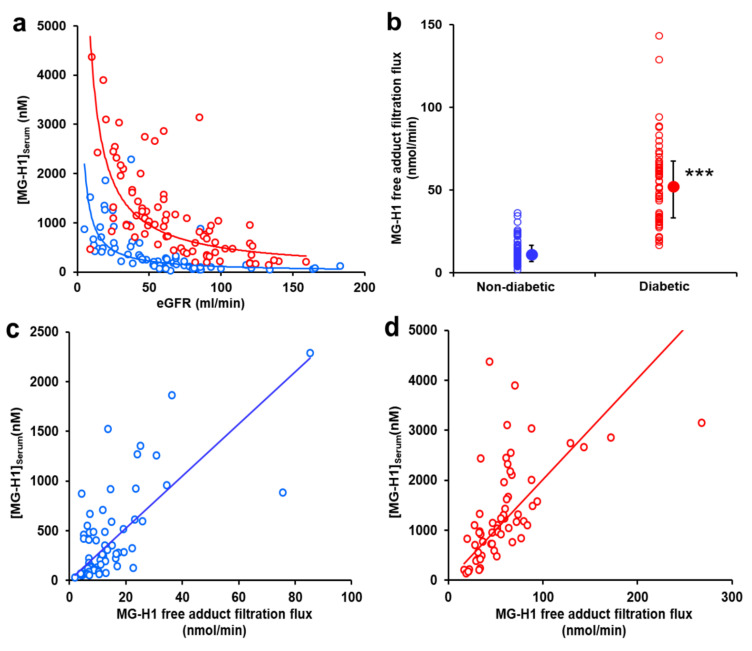
Dependence of serum methylglyoxal-derived hydroimidazolone MG-H1 free adduct concentration and filtration flux on estimated glomerular filtration rate and diabetes. (**a**) Plot of serum MG-H1 free adduct concentration against eGFR. Key: blue circle, control (subjects without diabetes); and red circle, subjects with diabetes. Hyperbolic regression line equations: non-diabetic—[MG-H1 free adduct]_Serum_ = 10,856 eGFR^−0.992^ (r = 0.75, n = 79); diabetic— [MG-H1 free adduct]_Serum_ = 37,658 × eGFR^−0.939^ (r = 0.70, n = 62). For eGFR as the dependent variable, the equations are: subjects without diabetes, eGFR = 968 x [MG-H1 free adduct]_Serum_^−0.560^, r = 0.74, *p* < 0.001); and subjects with diabetes, eGFR = 1838 × [MG-H1 free adduct]_Serum_^−0.515^, r = 0.70, *p* < 0.001). (**b**) MG-H1 glomerular filtration flux. Data are median (lower–upper quartile). Significance: ***, *p* < 0.001; *Mann-Whitney U test*. (**c**,**d**) Correlation of serum MG-H1 free adduct concentration with MG-H1 free adduct filtration flux. (**c**) Non-diabetic subjects and (**d**) diabetic subjects. Linear regression slopes are: (**c**) 26.2 ± 2.1 (r = 0.84; *p* < 0.001); and (**d**) 22.1 ± 1.5 (r = 0.84; *p* < 0.001).

AGE free adduct glomerular filtration flux (=eGFR × [AGE free adduct]_Serum_) was calculated for each AGE free adduct (Supplementary Data, Table S1). Most AGE free adduct filtration fluxes correlated positively with eGFR in subjects without or with diabetes; that is, AGE free adduct filtration flux declined with declining eGFR. An exception was MG-H1 free adduct filtration flux which did not correlate with eGFR for both subjects with and without diabetes. There was a strong inverse relationship between serum MG-H1 free adduct concentration and eGFR, with the hyperbolic curve describing the relationship, displaced to higher serum MG-H1 free adduct concentrations for subjects with diabetes (Figure 4a). Filtration flux of MG-H1 free adduct was increased in subjects with diabetes (Supplementary Data, Table S2). For normal renal function and all CKD stages combined, median MG-H1 filtration flux was increased 5-fold in subjects with diabetes, compared to subjects without diabetes (Figure 4b). There was no significant difference of MG-H1 filtration flux between subjects with type 1 and type 2 diabetes, male and female gender and absence or presence of hypertension or retinopathy.

In correlation analysis, for subjects without diabetes, MG-H1 filtration flux correlated positively with serum MG-H1 free concentration (r = 0.61, *p* < 0.001); and for subjects with diabetes, MG-H1 filtration flux correlated positively with both protein-bound MG-H1 (r = 0.34, *p* < 0.01) and serum MG-H1 free concentration (r = 0.70, *p* < 0.001) (Figure 4c,d). For subjects with diabetes, MG-H1 filtration flux correlated positively with age (r = 0.23, *p* < 0.05) but not with A1C or AER.

## 3. Discussion

AGEs are formed on proteins inside cells, proteins of the extracellular matrix and proteins of plasma, interstitial fluid and other body fluids (Figure 1). During protein turnover by cellular and extracellular proteolysis, AGE free adducts are formed and released into plasma. AGE free adducts pass readily through the glomerular filter and may be actively secreted and absorbed in the renal tubules with overall characteristic renal clearances and urinary excretion [3,15]. The urinary excretion of AGE free adducts reflects the flux of formation of AGEs in the body, corrected for contributions from the absorption of AGE free adducts formed by the digestion of AGE-modified proteins in ingested food. Proteins, mostly albumin, may leak through the glomerular filter. Albumin in the glomerular filtrate is mostly reabsorbed and transferred to the renal venous circulation by the albumin retrieval pathway with minor proteolysis and urinary excretion. Albumin and other proteins leak from the venous circulation to the interstitial fluid and are returned via the lymph system. The rate of leakage from the venous circulation is called the transcapillary escape rate (TER). Changes in albumin turnover, protein leakage through the glomerular filter and TER, as well as change in concentration of precursors of AGE formation and albumin, influence the steady-state level of AGE-modified proteins in serum. Changes in eGFR and renal clearance, as well as the flux of formation of AGE free adducts, influence the serum concentration of AGE free adducts, as recently reviewed [2].

In this study, we found that the most serum protein-bound AGEs were increased moderately and most serum AGE free adducts were increased markedly and progressively with decline in renal function through stages 2–4 CKD. The increase of serum protein MG-H1 and 3DG-H in patients with diabetes likely reflects the increased plasma concentrations of MG and 3-DG in diabetes and renal insufficiency [18,19,20,21,22], thereby increasing the in situ rate of glycation of plasma protein. Increased loss of albumin in CKD associated with albuminuria with compensatory increased albumin synthesis and turnover may counter increases in the steady-state level of serum protein-bound AGEs [23]. A progressive increase in serum AGE free adducts with decline in renal function in subjects with and without diabetes is expected as clearance from plasma is decreased [15]. The increased serum concentrations of MG-H1, 3DG-H and CMA free adducts in subjects with diabetes, compared to subjects with the same stage of CKD without diabetes, is consistent with increased rates of formation of AGEs in tissues and release into plasma after proteolysis in diabetes [24]. Overall, the analyte with greatest progressive increase from normal renal function to stage 4 CKD and increase in subjects with diabetes at all stages of CKD was MG-H1 free adduct. This analyte also was the AGE free adduct of highest serum concentration and readily and characteristically detected by LC-MS/MS as a double peak of epimers [3].

The accumulation of serum AGE free adducts in CKD is linked to decreased clearance with decline in eGFR [15,17]. If this is the dominant influence on serum AGE free adduct concentration, AGE free adduct glomerular filtration flux is expected to remain constant across the range of CKD stages studied. This was found in subjects with and without diabetes for MG-H1 only where there was no correlation of serum MG-H1 free adduct glomerular filtration flux with eGFR. Serum MG-H1 free adduct concentration increased markedly with decline in eGFR. Considering the mid-range of eGFR for stages 2–4 CKD, 75–22.5 mL/min/1.73 m^2^, from CKD-EPI and MDRD-4 equations for 50–70 year-old male and female subjects [25], serum creatinine concentration increases by 2.7–3.0 fold. For this range of eGFR, from the equations given (Figure 4a), serum MG-H1 increases 8.6 and 10.2-fold for subjects without and with diabetes, respectively. Hence, serum MG-H1 free adduct has a *ca.* 3-fold greater change over this range of eGFR than serum creatinine. MG-H1 free adduct had high renal clearance in subjects with normal renal function [15] and may have increased net tubular reabsorption as eGFR declines. Measurement of serum MG-H1 free adduct may provide an improved clinical indicator of eGFR as it is more sensitive to change in renal function than creatinine. This is deserving of further investigation.

MG-H1 free adduct glomerular filtration flux was increased *ca.* 5-fold in patients with diabetes, consistent with a profound increased exposure of the kidney to MG-H1 free adduct in diabetes independent of renal function status. This may reflect increased formation of MG-H1 in tissues in diabetes due to increased formation and accumulation of MG, as reviewed [26]. There may also be a contribution from increased absorption of MG-H1 free adduct from food due to increased intestinal permeability in diabetes [27], although collection of samples in the fasting state minimises this. The lack of correlation of MG-H1 free adduct glomerular filtration flux with A1C suggests it relates to factors other than glycemic control—such as the concentration of MG influenced by the rates of formation and metabolism of MG—the latter occurring mainly through activity of glyoxalase 1 (Glo1) of the glyoxalase system [28]. The positive correlation of MG-H1 free adduct glomerular filtration flux with subject age may reflect the age-dependent decline in activity of Glo1 [28]. Increased MG concentration in diabetes which may be linked to increased progression of CKD [26].

For other AGEs, AGE free adduct glomerular filtration flux decreased with decreasing eGFR; that is, the increase in serum AGE free adduct concentration with decline in renal function is less than expected for the loss of clearance, as found for other non-AGE metabolites in CKD [29]. This effect may be due to increased fractional excretion of AGE free adducts, linked to decreased tubular re-uptake of the AGE free adduct in advanced stages of CKD. Indeed, we found that increased fractional excretion of AGE free adducts occurs in subjects with type 1 diabetes and microalbuminuria who later progress to early decline in renal function. Fractional excretion of AGE free adducts may increase in subjects with stages 2–4 CKD [30]. AGE free adducts are thought to be reabsorbed by lysine and arginine transporters of renal proximal tubules [30] and, interestingly, genome-wide association studies identified linkage of genetic polymorphisms of these transporters to decline in GFR [31,32].

An unexpected finding was a lack of increase in protein-bound CML and G-H1 in subjects with diabetes, compared to non-diabetic, including in some stages of CKD. CML is formed partly from glyoxal and G-H1 is formed exclusively from glyoxal, a product of lipid peroxidation [16,33]. Differences in lipid peroxidation status may contribute to this finding. Serum pentosidine free adduct concentration was also remarkably higher in subjects with normal renal function without diabetes than with diabetes. The higher age of subjects without diabetes compared to subjects with diabetes (mean age 67 vs. 43 years; *p* < 0.01) may explain this. In older subjects (50% over 70 years old), it is likely that there was a high prevalence of early-stage osteoarthritis [34]. Plasma pentosidine free adduct is increased in subjects with early-stage osteoarthritis linked to increased proteolysis of joint proteins [35].

Limitations of this study are the relatively small subject number in some study groups, younger age of subjects with diabetes and normal renal function compared to other study groups, and poor gender balance in some study groups. However, the magnitudes of the changes found, particularly for AGE free adducts, and limited previous studies characterising changes in serum AGE free adducts in CKD, provide an advance in understanding in renal handling of AGEs in stages 2–4 CKD.

## 4. Materials and Methods

### 4.1. Patients and Study Design

This study was a cross-sectional survey of patients attending the diabetes clinic at Austin Health, a tertiary referral center and teaching hospital of the University of Melbourne, Victoria, Australia. Informed consent was obtained from patients, as approved by the Austin Health Human Research Ethics Committee (Project numbers H2005/02118 and H2003/01642). Study participants had or did not have diabetes and were in 4 categories of eGFR: 15–30, 31–60 and 61–90 mL/min/1.73 m^2^ (stages 4, 3 and 2 CKD, respectively) and >90 mL/min/1.73 m^2^ with no indication of functional or structural damage (proteinuria, glomerulonephritis, polycystic kidneys)—subjects with normal renal function; n = 7–29 per category. For patients with diabetes, presence of diabetic kidney disease was considered on the basis of long-standing duration of diabetes, preferably presence of retinopathy (53% of subjects recruited) and gradual loss of eGFR in the absence of known non-diabetic kidney disease [36]. However, it should be noted that the presence of retinopathy is now not considered an obligatory manifestation of diabetic kidney disease, especially in the setting of good metabolic control, use of renin-angiotensin blockers, and the association with micro- or normoalbuminuria [37,38]. Fasting serum samples were obtained prior to a routine clinic visit and stored at −20 °C. Samples were transferred on dry ice to the collaborating laboratory in the UK and stored at −80 °C until analysis. Urinary albumin was measured as described previously [39]. Creatinine was measured on an automatic analyzer and eGFR was calculated using the MDRD-4 formula.

### 4.2. Analysis of Serum Protein-Bound AGEs and AGE Free Adducts

Protein-bound AGEs were quantified in exhaustive enzymatic digests of serum protein by stable isotopic dilution analysis liquid chromatography-tandem mass spectrometry (LC-MS/MS), with correction for autohydrolysis of hydrolytic enzymes as described [40]. AGE free adducts were determined in ultrafiltrates (10 kDa cut-off) of the same samples. AGEs determined were CML; N_ε_-(1-carboxyethyl)lysine (CEL); hydroimidazolones derived from glyoxal, methylglyoxal and 3-deoxyglucosone (G-H1, MG-H1 and 3DG-H, respectively); N_ω_-carboxymethylarginine (CMA); pentosidine and glyoxal and methylglyoxal-derived lysine dimers (GOLD and MOLD, respectively); and amino acids—arginine, lysine and valine in hydrolysates (valine content is required for the protease autohydrolysis correction) [40]. Protein-bound AGEs were normalised to arginine or lysine residue precursors and given as mmol/mol amino acid modified; and related free adducts were given in nM. Chemical structures and clinical significance of AGE analytes are described elsewhere [2,41].

### 4.3. Statistical Analysis

Data are mean ± SD for parametric data and median (upper–lower quartile) for non-parametric data. Significance testing was by paired Student’s t-test and Mann-Whitney U test (for 2 groups), by one-way ANOVA and Kruskal-Wallis test (for 4 groups) for parametric and non-parametric data, respectively, and correlation analysis by the Spearman method. Statistical analyses were performed using SPSS (version 24.0, Armonk, NY, USA).

## 5. Conclusions

Serum MG-H1 free adduct was the AGE in serum with greatest progressive change in concentration in subjects with decline in renal function through to stage 4 CKD. Serum MG-H1 free adduct concentration was highly and inversely linked to eGFR. Glomerular filtration flux of MG-H1 free adduct—reported herein for the first time—was increased 5-fold in patients with diabetes, independent of stage of CKD. Serum MG-H1 free adduct concentration may be a strong risk marker of CKD in subjects with and without diabetes with diagnostic advantages over the current reference clinical chemistry analyte, creatinine. From this study it may be inferred that a simple measurement of serum MG-H1 free adduct in clinical practice may improve the assessment of temporal change in eGFR and thereby improve monitoring of the trajectory of decline in renal function at all stages of CKD in diabetic kidney disease.

## Figures and Tables

**Table 1:** Clinical characteristics of study participants.

CKD Stage	Diabetes Status	Normal	2	3	4	*p*-Value
Number of subjects	No	12	20	16	25	
	Yes	7	15	29	11	
Age (years)	No	67 ± 17	72 ± 13	80 ± 11 *	76 ± 14	
	Yes	43 ± 11 ^OO^	65 ± 9 ***	74 ± 11 ***	76 ± 15 ***	<0.001
Gender (M/F)	No	4/8	6/14	12/4	20/5	
	Yes	0/7	13/2	17/12	7/4	
Diabetes type (N for types 1 and 2)	Yes	3,4	0,15	3,26	0,11	
Duration of diabetes (years)	Yes	13 ± 9	16 ± 8	20 ± 12	18 ± 9	
A1C (%)	Yes	7.9 ± 1.0	7.6 ± 0.8	7.9 ± 1.3	8.2 ± 1.3	
eGFR (ml/min)	No	130 ± 41	73 ± 11 **	49 ± 14 ***	21 ± 10 ***	<0.001
	Yes	126 ± 20	70 ± 14 ***	46 ± 15 ***	26 ± 11 ***	<0.001
Albumin excretion rate (mg/24 h)	No	NA	NA	NA	NA	
	Yes	24 (17–36)	13 (12–92)	53 (20–106)	225 (44–722)**	<0.01

Data are proportions, mean ± SD or median (lower–upper quartile). Significance: comparison of all 4 groups—*p*-value shown; comparison of two groups—*, ** and ***, *p* < 0.05, *p* < 0.01 and *p* < 0.001 with respect to subjects with normal renal function of the same diabetes status, and ^OO^, *p* < 0.01, with respect to subjects without diabetes of the same renal function status. Abbreviation: NA, data not available.

## Data Availability

The data presented in this study are available upon request from the corresponding author.

## Supplementary Materials

Supplementary data are available online: https://www.mdpi.com/article/10.3390/ijms24010152/s1.
