# Supplementary material for: Analysis of Serum Advanced Glycation Endproducts Reveals Methylglyoxal-Derived Advanced Glycation MG-H1 Free Adduct Is a Risk Marker in Non-Diabetic and Diabetic Chronic Kidney Disease
Source: Int J Mol Sci. 2022 Dec 21;24(1):152. doi: 10.3390/ijms24010152 (PMC9820473; doi:10.3390/ijms24010152)
Supplement: Supplementary file 1 [file ijms-24-00152-s001.zip › ijms-2061197-supplementary.pdf]

Supplementary data

Table S1. Correlation of serum advanced glycation endproducts with age, eGFR and filtration flux.

AGE	Protein-bound AGE vs subject age		AGE free adduct vs subject age		Protein-bound AGE vs eGFR		AGE free adduct vs eGFR		AGE free adduct filtration flux vs eGFR	
	Non-diabetic	Diabetic	Non-diabetic	Diabetic	Non-diabetic	Diabetic	Non-diabetic	Diabetic	Non-diabetic	Diabetic
CML				0.49***	- 0.42**	- 0.38**	- 0.69***	- 0.73***	0.57***	0.42***
CEL		0.42***		0.36**		- 0.50***	- 0.80***	- 0.58***	0.41**	0.76***
G-H1				0.48***			- 0.80***	- 0.61***		0.49***
MG-H1				0.54***	- 0.48***	- 0.31**	- 0.79***	- 0.74***		
3DG-H				0.46***	- 0.39*	- 0.32*	- 0.80***	- 0.66***	0.46**	0.61***
CMA				0.35*	- 0.38*		- 0.81***	- 0.50***		0.52***
GOLD									0.86***	0.62***
MOLD					- 0.44**		- 0.44**		0.52***	0.73***
Pentosidine		0.44***		0.58***		- 0.52***	- 0.73***	- 0.66***	0.40**	

Spearman correlation coefficients for eGFR and AGEs. Significance: *, ** and ***, P<0.05, P<0.01 and P<0.001 (Bonferroni correction of 9 applied).

Table S2. Glomerular filtration flux of AGE free adducts.

AGE	Control		Stage 2 CKD		Stage 3 CKD		Stage 4 CKD	
	Non-diabetic	Diabetic	Non-diabetic	Diabetic	Non-diabetic	Diabetic	Non-diabetic	Diabetic
CML	19.7 (18.7 – 33.1)	6.9 (6.4 – 7.6) *	9.7 (8.4 – 12.9) ^{OO}	7.2 (5.7 – 9.7)	9.5 (6.8 – 11.3) ^{O}	6.2 (4.4 – 8.2) **	7.5 (5.7 – 11.7) ^{O}	5.2 (4.2 – 7.2)
CEL	10.0 (7.9 – 18.4)	18.1 (14.1 – 18.9)	7.1 (4.9 – 8.7)	9.0 (7.6 – 10.5) *^{,OOO}	7.5 (6.0 – 8.7)	6.8 (5.8 – 9.0) ^{OOO}	5.5 (3.8 – 11.8)	7.0 (4.9 – 8.8) ^{OO}
G-H1	11.1 (7.8 – 23.1)	5.5 (4.0 – 6.7)	6.6 (4.8 – 8.9)	4.0 (2.8 – 4.6) ***	9.0 (6.5 – 10.6)	4.2 (3.0 – 5.6) ***	7.5 (4.5 – 10.6)	3.2 (1.7 – 6.1)
MG-H1	10.4 (6.7 – 15.9)	22.1 (21.3 – 33.5) *	8.7 (4.6 – 16.4)	48. 4 (31.2 – 67.7) ***	12.1 (11.0 – 14.9)	58.7 (38.5 – 78.3) ***^{,OO}	12.6 (6.9 – 24.1)	62.0 (43.7 – 70.2) ***^{,OO}
3DG-H	4.7 (3.8 – 5.3)	7.5 (6.4 – 8.1) *	3.1 (2.8 – 3.5)	5.9 (4.2 – 6.6) ***	3.2 (2.4 – 3.8)	4.9 (3.8 – 6.9) *	2.9 (1.6 – 4.6)	4.5 (3.6 – 7.7)
CMA	1.6 (1.0 – 2.2)	3.0 (2.0 – 3.6)	1.1 (0.8 – 1.5)	1.9 (1.6 – 3.1) *	1.2 (1.1 – 1.4)	2.0 (1.6 – 3.2) ***	2.0 (0.9 – 3.0)	3.0 (1.8 – 3.4)
GOLD	0.039 (0.033 – 0.045)	0.060 (0.019 – 0.090)	0.022 (0.013 – 0.025) ^{O}	0.022 (0.015 – 0.033)	0.019 (0.014 – 0.022) ^{OO}	0.011 (0.005 – 0.020) ^{OO}	0.004 (0.002 – 0.008) ^{OOO}	0.013 (0.006 - 0.016)**^{,O}
MOLD	0.273 (0.159 – 0.478)	0.101 (0.062 – 0.178)	0.137 (0.075 – 0.195)	0.077 (0.059 – 0.135)	0.102 (0.073 – 0.173)	0.051 (0.034 - 0.069) **^{,O}	0.095 (0.050 – 0.127) ^{O}	0.032 (0.025 – 0.046)*^{,OO}
Pentosidine	0.090 (0.075 – 0.121)	0.015 (0.012 – 0.024) **	0.042 (0.036 – 0.053) ^{OO}	0.023 (0.016 – 0.035) *	0.048 (0.036 – 0.065)	0.032 (0.017 – 0.062)	0.041 (0.025 – 0.072)	0.050 (0.037 – 0.052) ^{OO}

Data are median (lower – upper quartile) nmol/min (n = 7 – 29). Significance: *, ** and ***, P<0.05, P<0.01 and P<0.001, with respect to subjects with the same CKD status without diabetes and o, oo and ooo, P<0.05, P<0.01 and P<0.001, with respect to subjects with normal renal function and the same diabetes status; *Mann-Whitney U test* (Bonferroni correction of 9 applied). Cross-group comparison (P value): non-diabetic CML, 0.039 and GOLD, 1.8 x 10^{-8} and diabetic CEL, 0.0012, GOLD, 0.016 and MOLD, 0.0013; *Kruskal-Wallis*. Bonferroni correction of 9 applied.
